# Endoscopic detection of signet ring cell carcinoma in *CDH1* carriers: a 15-year single-centre experience

**DOI:** 10.1007/s10689-026-00586-9

**Published:** 2026-07-06

**Authors:** Omar Salehi, Douglas Tjandra, Jadon Karp, Alex Boussioutas

**Affiliations:** 1 Familial Cancer Clinic, Peter MacCallum Cancer Centre, Melbourne, VIC 3000 Australia; 2 Department of Gastroenterology, Alfred Health, Melbourne, VIC 3004 Australia; 3 School of Translational Medicine, Monash University, Melbourne, 3004 Australia; 4 Department of Medicine, University of Melbourne, Parkville, 3010 Australia

**Keywords:** *CDH1*, Hereditary diffuse gastric cancer, Signet ring cell carcinoma, Endoscopic surveillance, Gastrectomy

## Abstract

*CDH1* pathogenic variant carriers are at high lifetime risk of hereditary diffuse gastric cancer (HDGC). Endoscopic surveillance is recommended for individuals who delay risk-reducing total gastrectomy, although detection of signet ring cell carcinoma (SRCC) remains challenging. No Australian cohort has reported real-world endoscopic performance with surgical correlation in this population. We performed a 15-year retrospective cohort study of adult *CDH1* carriers undergoing esophagogastroduodenoscopy (EGD) at a tertiary familial cancer centre (2008–2023). Patients presenting with symptomatic invasive gastric cancer at initial endoscopy were excluded. Most EGDs were conducted as preoperative assessments prior to risk-reducing total gastrectomy, with a minority undertaken as longitudinal surveillance. Endoscopic findings, biopsy performance, surgical pathology, and postoperative outcomes were analysed. Eighty-two *CDH1* carriers from 31 families underwent 136 EGDs (median 1 per patient). SRCC was detected endoscopically in 38 patients (46.3%), predominantly via random biopsies. Overall sensitivity for SRCC detection was 67.9%, with random biopsies outperforming targeted sampling. Most lesions were identified at the initial EGD. Sixty-nine patients (84.1%) proceeded to total gastrectomy; SRCC was confirmed in 56 (81.2%), with variable tumour burden (1–73 foci). Invasive disease beyond pT1a was uncommon (2.9%). Postoperative complications occurred in 43.5%, most frequently anastomotic strictures requiring dilatation; there were no procedure-related deaths. Although occult SRCC was common, invasive malignancy beyond pT1a was infrequent, suggesting that many intramucosal lesions may have a more indolent course than previously assumed. Detection of SRCC at endoscopy remains challenging; however, these findings support a more individualized, risk-adapted approach to *CDH1* management. Longer-term prospective data are required to better define SRCC progression risk and guide decisions regarding surveillance and risk-reducing surgery.

## Introduction

Hereditary diffuse gastric cancer (HDGC) is an autosomal dominant cancer predisposition syndrome caused by pathogenic variants in the *CDH1* gene, which encodes the cell adhesion protein, E-cadherin [1–3]. *CDH1* pathogenic variants have historically been associated with a high lifetime risk of diffuse gastric cancer, reported up to 70–83% by age 80; however more recent data suggests that these estimates may be influenced by ascertainment bias, and may be as low as 7–10% in less selected cohorts, demonstrating that cancer risk is highly variable between families [4–6]. *CDH1* pathogenic variants are also associated with an increased risk of lobular breast cancer (LBC) and lobular carcinoma in situ (LCIS), with cumulative risk up to 55% [6, 7].

International guidelines recommend risk reducing total gastrectomy for *CDH1* carriers, typically between ages 20–30 years, particularly those with family history of diffuse gastric cancer [2]. Although effective for cancer risk reduction, gastrectomy carries substantial morbidity and lifelong nutritional consequences, creating a complex decision for young, asymptomatic carriers [8–12]. For those who delay or decline surgery, structured endoscopic surveillance is advised [2].

The histological hallmark of HDGC is intramucosal signet ring cell carcinoma (SRCC), which typically presents as microscopic foci without visible mass lesions [13]. Endoscopic protocols have evolved from limited random biopsies to systematic mapping strategies such as the Cambridge Protocol and subsequent high-intensity modifications [14, 15]. Despite these advances, reported detection rates remain variable, ranging from 15–67% across series [14–19].

We conducted a retrospective analysis of endoscopic detection of SRCC in *CDH1* carriers undergoing esophagogastroduodenoscopy (EGD) at our Australian tertiary familial cancer centre over a 15-year period, representing the first Australian cohort, to our knowledge, to report real-world endoscopic detection rates with surgical pathology correlation in *CDH1* carriers. Historically, most endoscopies were performed as preoperative assessment prior to risk reducing total gastrectomy rather than as part of longitudinal surveillance, reflecting earlier guideline emphasis on definitive surgery, although contemporary practice is shifting toward increased uptake of endoscopic surveillance. This distinction is important when interpreting detection rates.

The primary objective was to assess endoscopic performance for SRCC detecting using surgical pathology as the reference standard. Secondary objectives included evaluating biopsy strategy performance, surgical outcomes and surveillance patterns to inform future clinical decision making in *CDH1* carriers.

## Methods

This single-centre retrospective cohort study was conducted at a tertiary familial cancer centre between January 2008 and December 2023. Ethics approval was obtained from the institutional research ethics board (Project ID: QA/97246/PMCC).

We included adults (≥ 18 years) with confirmed pathogenic or likely pathogenic (Class 4 or 5) *CDH1* variants [21] who underwent at least one EGD at our institution. Patients with variants of uncertain significance or insufficient follow-up were excluded. Those who had symptomatic disease of invasive gastric cancer at initial endoscopy were excluded from the analytic cohort, as these represent index cases rather than individuals undergoing surveillance.

During the study period, EGD primarily served as a preoperative evaluation prior to upfront risk reducing total gastrectomy, consistent with international guideline recommendations for most of the study period, with a smaller subset declining upfront surgery and opting for endoscopic surveillance annual surveillance.

EGDs were performed predominantly by gastroenterologists experienced in hereditary gastrointestinal cancer syndromes. A small number of EGDs were conducted at external sites for which endoscopic and pathological reports were obtained and reviewed when possible.

The majority of endoscopies were performed by gastroenterologists experienced in hereditary gastrointestinal cancer syndromes at our institution. In our study, we generally utilized the HDGC surveillance protocol which includes systematic sampling with 28–30 random biopsies from key stomach regions (cardiac, fundus, body, transitional zone, antrum) as well as targeted biopsies from mucosal lesions. A small number of procedures were performed at external centers, all biopsy specimens were processed according to standard protocols and examined by experienced pathologists. Histological assessment included evaluation for SRCC and other pathological findings.

Biopsy specimens were processed using standard protocols and reviewed by experienced pathologists for SRCC and other abnormalities. Patients proceeding to total gastrectomy underwent standardised surgical resection by specialist upper gastrointestinal surgeons. Gastrectomy specimens were systematically sectioned and examined histologically, with tumour burden quantified by counting individual foci by the reporting pathologist.

To compare rates of *Helicobacter pylori* infection in the non-*CDH1* population, a separate cohort of Australian-born individuals, evaluated over a similar time period and using comparable methodology (21), was utilised from our research group (Project 430/23/AH).

### Data collection

Demographic, genetic, endoscopic, surgical, and pathological data were extracted from electronic medical records using a standardised collection tool.

### Statistical analysis

Categorical variables were summarised as frequencies and percentages, and continuous variables as means (SD) or medians (IQR) as appropriate. Group comparisons were performed using chi-square or Fisher’s exact tests for categorical variables and Student’s t-test or Mann–Whitney U test for continuous variables. Univariate analysis was used to assess factors associated with SRCC detection, with *p* < 0.05 considered statistically significant. Analyses were conducted using GraphPad Prism (v10.5.0).

## Results

### Cohort characteristics

A total of 90 patients with *CDH1* Class 4 or 5 pathogenic variants were identified. Six patients were excluded due to lack of data and/or incomplete follow up. Two additional patients who presented with symptomatic invasive gastric cancer at initial endoscopy were excluded from the analytic cohort.

Eight-two patients who were *CDH1* pathogenic variants and underwent at least one EGD were included in the study cohort. The cohort comprised patients from 31 unique families.

Six patients (7.3%) had *Helicobacter pylori* detected on biopsy. In comparison with a contemporaneous Australian-born non-*CDH1* cohort (*n* = 456) from our research group, CDH1 carriers had a significantly lower prevalence of *H. pylori* on biopsy (7.3% vs. 24.1%; *p* < 0.001), which remained significant after adjustment for age (adjusted OR 0.33, 95% CI 0.14–0.78, *p* = 0.011).

The remaining baseline characteristics are summarised in Table 1.

**Table 1:** Cohort demographics

Total patients *N*	82
Number of families *N*	31
Age (median, IQR)	45 (33.75–58.75)
Female *N* (%)	51 (62.2%)
**Smoker N (%)**	
Current	11 (13.4%)
Former	9 (11%)
Never	60 (73.2%)
Unknown	2 (2.4%)
*Helicobacter pylori* detected on biopsy N (%)	6 (7.3%)
**Family history HDGC N (%)**	
First degree relative	71 (86.6%)
Non first degree relative only	8 (9.8%)
No known family history	3 (3.7%)
**Died N (%)**	
Total	4 (4.9%)
Metastatic disease	3 (3.7%)
Unclear cause	1 (1.2%)

### Endoscopic detection and surgical correlation

**Fig. 1:**
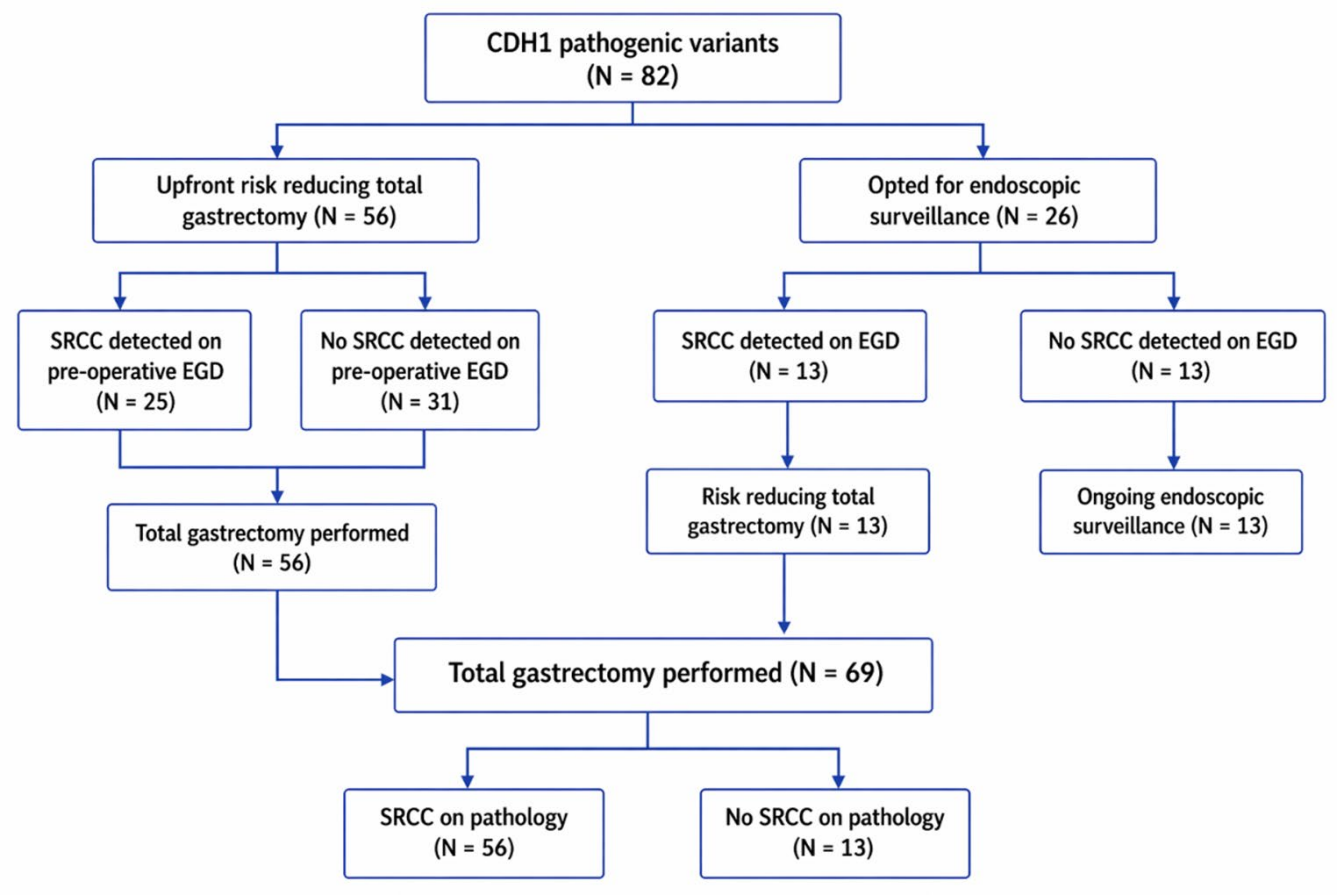
Patient flowchart

Management pathways are of the 82 *CDH1* pathogenic variant carriers included in the cohort illustrated in Fig. 1.

Total gastrectomy was performed in 69 (84.1%) patients at a median age of 37 years (range 19–66). Fifty-six (68.3%) patients elected to undergo upfront surgery while 26 patients initially opted for endoscopic surveillance.

Among the 56 patients who underwent upfront risk reducing total gastrectomy, SRCC was detected on routine pre-operative EGD in 25 patients (44.6%), while 31 (55.4%) patients did not have SRCC detected prior to surgery.

Among the 26 patients initially undergoing surveillance, SRCC was detected on EGD in 13 patients (50.0%) all of whom proceeded to risk reducing total gastrectomy, while 13 (50.0%) remain under ongoing endoscopic surveillance. Of the 69 total patients who underwent gastrectomy, surgical histopathology demonstrated SRCC in 56 (81.2%).

Endoscopic detection of SRCC is summarised in Table 2. A total of 136 EGDs were performed across the cohort (range 0–8 per patient, median 1). Using gastrectomy histopathology as the reference standard, overall sensitivity of EGD for SRCC detection was 67.9% (38/56), with random biopsies contributing to 58.9% (33/56), compared with 8.9% (5/56) for targeted biopsies. Across all 136 EGDs, 2720 random biopsies yielded 33 SRCC-positive samples (per-biopsy positivity 1.2%), whereas 5 of 64 targeted biopsies were SRCC-positive (per-biopsy positivity 7.8%).

**Table 2:** Endoscopic detection of SRCC

Total number of EGDs	136
Median EGDs per patient (range)	1 (0–8)
**Number of EGDs per patient N (%)**	
1	51 (62.2%)
≥ 2	31 (37.8%)
**Patients with SRCC detected on endoscopy N**	
Total	38
Random biopsy	33
Targeted biopsy	5
**Sensitivity**	
Overall	67.9% (38/56)
Random biopsy	58.9% (33/56)
Targeted biopsy	8.9% (5/56)
**Per-biopsy detection**	
Random biopsy	1.2% (33/2720)
Targeted biopsy	7.8% (5/64)

**Fig. 2:**
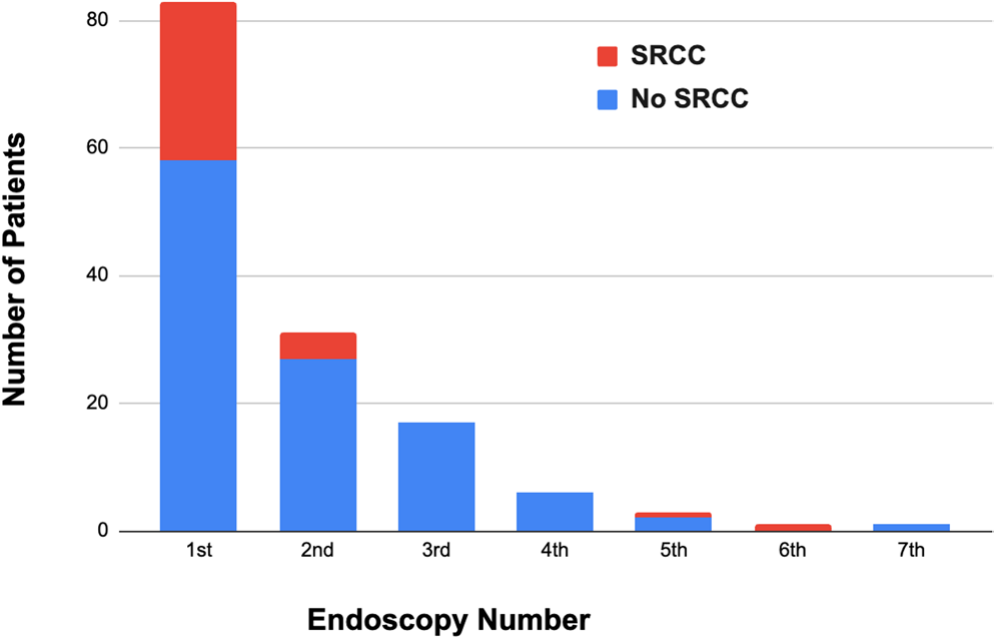
Sequential endoscopic SRCC detection

Analysis of sequential endoscopies (Fig. 2) demonstrated that most SRCC detections occurred during the first endoscopic examination (30%, 25/83 procedures). Detection rates declined substantially in subsequent endoscopies, although isolated cases were identified as late as the fifth and sixth surveillance EGDs in individuals with multiple prior negative examinations.

### Macroscopic abnormalities

Sixty-six (48.5%) of the 136 EGDs documented macroscopic mucosal abnormalities. No specific visible lesion was predictive of SRCC. Pale mucosal areas were identified in 24 cases, with SRCC detected in 4 specimens (16.7%, *p* = 0.2157). Nodular mucosal lesions were present in 6 cases (29.9%), with none corresponding to SRCC detection (*p* = 0.1827). Erosions or ulcers were found in 15 cases with SRCC detected in only 2 (13.3%, *p* = 0.2317). Polyps demonstrated a statistically significant negative association with SRCC detection, with 0/13 polyps harboring malignancy (*p* = 0.0197). Erythematous lesions were observed in 8 cases with SRCC detected in only 1 (12.5%, *p* = 0.4395).

**Fig. 3:**
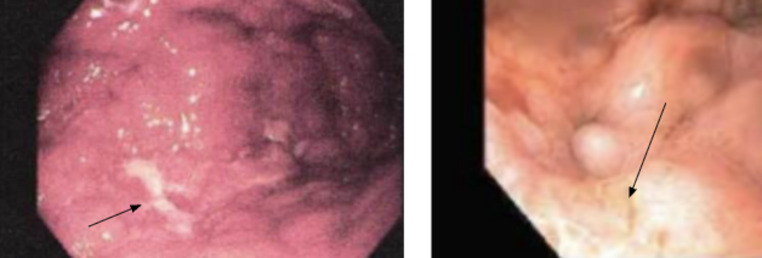
Invasive Lesions in *CDH1* carriers. **Left**: Invasive gastric cancer demonstrating erythematous, irregular mucosa with focal ulceration (arrow). **Right**: Invasive gastric cancer demonstrating pale, ulcerated changes (arrow)

Invasive lesions (> pT1a), were associated with visible endoscopic abnormalities **(**Fig. 3) and were recognized as suspicious at the time of endoscopy.

### Surgical outcomes

In our cohort of 69 patients who underwent total gastrectomy, SRCC was detected in 56 (81.2%) patients. The median number of tumor foci was 5 per patient (range 1–73). Invasive malignancy (> pT1a) at diagnosis was present in 2 (2.9%) patients. The median length of hospital stay was 4 days (range 3–27 days).

SRCC anatomical distribution most commonly involved the fundus (39.4%), followed by the antrum (33.0%) and body (30.3%). Involvement of the cardia and transitional zone was less frequent (10.6% in each). Given the presence of multifocal disease, percentages exceed 100%.

Post-operative complications **(**Table 3**)** occurred in 30/69 patients (43.5%), with 21/69 (30.4%) requiring additional procedures within 90 days; most commonly endoscopic dilation of anastomotic stricture. 6 patients (8.7%) had a complication within 30 days, 10 patients (14.5%) between 30 and 90 days and 14 patients (20.3%) after 90 days.

**Table 3:** Post operative complications

Timeframe	Complication (number of patients, %)
≤ 30 days	**Total 6/69 (8.7%)** PE/DVT: 2 (2.9%) Ileus: 1 (1.4%) Anastomotic leak managed conservatively: 1 (1.4%) Surgical wound infection managed with antibiotics: 1 (1.4%) Hospital acquired pneumonia managed with antibiotics: 1 (1.4%)
30–90 days	**Total 10/69 (14.5%)** Anastomotic stricture requiring endoscopic dilation: 8 (11.6%) Anastomotic leak requiring return to theatre: 1 (1.4%) DVT: 1 (1.4%)
> 90 days	**Total 14/69 (20.3%)** Anastomotic stricture requiring endoscopic dilation: 10 (14.5%) Small bowel obstruction due to adhesion managed with adhesiolysis: 2 (2.9%) Incisional hernia–surgically repaired: 1 (1.4%) Paraoesaphageal hernia–surgically repaired: 1 (1.4%)

### Lobular breast cancer and SRCC

From the 31 families in the study, 33/82 patients (40.2%) had a family history of breast cancer, with 30 patients (36.6%) having a documented history of lobular breast cancer and/or LCIS. An additional 3 patients (3.7%) reported a family history of breast cancer without clear documentation of subtype.

Nine patients (11.0%) had a personal history of lobular breast cancer, 7 (8.5%) of whom had at least one focus of SRCC detected on endoscopy or surgical pathology. Two patients (2.4%) had a history of LCIS, 1 (1.2%) of whom had SRCC detected on surgical pathology. No patients had > T1a lesions detected.

In two cases, a diagnosis of lobular breast cancer led to genetic testing, and these patients were the probands for identification of the *CDH1* pathogenic variant. Both were subsequently found to have SRCC detected on endoscopic surveillance and proceeded to total gastrectomy.

## Discussion

In this retrospective observational cohort study—the first to evaluate endoscopic performance in 82 *CDH1* carriers within an Australian centre—we report real-world SRCC detection rates and surgical correlation over a 15-year period.

Most SRCC detections occurred during the first EGD, with substantially lower yield on subsequent procedures. This pattern suggests that most detectable lesions are identified early in the surveillance process [18]. The declining yield over time likely reflects both the underlying biology of *CDH1*-associated SRCC, characterised by intramucosal foci present from a young age, and the technical limitations of surveillance, whereby patients with repeatedly negative examinations may harbour microscopic lesions below current detection thresholds.

While diminishing yield after multiple negative examinations may suggest reduced incremental benefit of frequent surveillance [14, 20], the continued detection of SRCC in later procedures, including isolated cases at 5th and 6th endoscopies, supports the importance of maintaining surveillance programs for patients who opt against risk-reducing surgery.

With respect to the clinical context of our cohort: for much of the study period, guideline recommendations and counselling strongly favoured risk reducing gastrectomy, particularly following identification of SRCC [2, 22, 23]. Consequently, most EGDs were performed as preoperative assessments prior to risk reducing surgery rather than structured longitudinal surveillance. Over the past decade, however, data highlighting the ubiquity of pT1a lesions, the potential risk of overtreatment—especially in younger patients—and the low incidence of advanced cancer during surveillance have contributed to a more individualised, risk-adapted approach [16, 24, 25].

In the context of our cohort, among the 71 patients undergoing total gastrectomy, SRCC was present in 81.7% of specimens; however, invasive malignancy beyond pT1a was identified in only 2 patients (2.9%). Thus, although occult SRCC was common, progression to deeper invasive disease at the time of surgery was uncommon. Emerging surveillance cohorts have similarly shown low rates of progression to advanced gastric cancer, even among individuals with biopsy-proven intramucosal SRCC [16]. Our data support the growing recognition that many non-targeted, intramucosal SRCC lesions may be more indolent than previously assumed. These findings add to the contemporary perspective, with increasing acceptance of endoscopic surveillance as a reasonable option for selected patients rather than upfront risk reducing surgery.

With regards to endoscopic biopsy sensitivity, our cohort had an overall sensitivity of 67.9% for endoscopic SRCC detection, with a higher rate of detection in random biopsies than targeted biopsies (58.9% vs. 8.9%). The inherent biology of SRCC lesions make them difficult to detect as they typically manifest as microscopic foci without distinct macroscopic features, with previous models estimating that approximately 1700 biopsies would be required to achieve a 90% probability of detecting at least a single SRCC focus, highlighting the inherent limitations of endoscopic surveillance [23].

Studies utilising the earlier HDGC surveillance biopsy protocol have reported detection rates of 15–61% [9, 15, 18, 26, 27]. More recent approaches incorporating systematic and more extensive biopsy sampling have demonstrated improved diagnostic performance. The Lee et al. 16-year prospective study achieved 67.3% sensitivity in *CDH1* carriers using a modified Cambridge Protocol [14] while Asif et al., demonstrated a 63% SRCC detection rate using the Bethesda protocol [16]. Our overall detection rate of 67.9% is largely consistent with the literature and reflects the contribution of systematic random biopsy protocols to the detection of occult SRCC.

However, the clinical utility of detecting isolated superficial pT1a SRCC lesions is uncertain. Current guidelines underscore that surveillance aims to exclude higher-risk disease rather than detect every microscopic focus (2). In this setting, random biopsies remain important in surveillance of patients deferring gastrectomy, although repeated sampling may also result in mucosal scarring that could potentially obscure subsequent SRCC lesions (18,28).

Furthermore, while intramucosal SRCC is frequently identified, the ability to distinguish lesions that will progress to clinically significant disease remains suboptimal, acknowledging that the risk of invasion is likely lower than previously thought and upfront risk reducing gastrectomy can safely be deferred or avoided. However, this is an important point in counselling of patients of the risk of developing an advanced diffuse gastric cancer. For some patients, the finding of an SRCC even if indolent is enough motivation to lead to risk reducing surgery and this is an important patient decision.

Interestingly, when calculated per biopsy, SRCC was detected in 1.2% of random and 7.8% of targeted samples. Although the per-biopsy yield of targeted biopsies was higher in targeted biopsies, these represented a minority percentage (2.4%) of total endoscopic biopsies performed; thus, the overall diagnostic contribution of targeted sampling remained limited. Of note, this finding is consistent with prior studies (18,29) which suggest that although targeted biopsies are fewer in number, they may contribute disproportionately to SRCC detection, while extensive random sampling yields a low per-biopsy diagnostic rate. Overall, these findings highlight the complementary roles of both targeted and random biopsy strategies, with careful mucosal inspection guiding targeted sampling, while systematic random biopsies may still detect otherwise occult lesions.

Macroscopic abnormalities have traditionally been considered potential sites of malignant change [2, 15, 16, 18, 28], particularly pale mucosal areas [14, 20]. In our study, while 4 of 24 (16.7%) documented pale areas detected SRCC, this did not reach statistical significance. Polyps demonstrated a significant negative association with SRCC detection, consistent with prior studies reporting SRCC detection rates within polyps of 0–1.8% [14, 16, 20], without a clear significant positive association. This is consistent with our findings and corroborates that SRCC are rarely found in polyps.

Importantly, the two cases of invasive disease (> pT1a) in our cohort were associated with visible mucosal abnormalities and were recognised as suspicious at the time of endoscopy, with representative endoscopic images shown (Fig. 3). This emphasises that, while detection of occult intramucosal SRCC may be imperfect and of uncertain clinical consequence, careful endoscopic inspection remains critical for the identification of lesions with features suggestive of more advanced disease.

The low prevalence of *H. pylori* observed in our cohort is broadly consistent with the limited published HDGC surveillance literature (14,16,19), which has suggested that CDH1 carriers may be at lower risk of *H. pylori* infection. This difference should also be interpreted in the context of ascertainment bias and age, as non-*CDH1* comparator cohorts undergoing endoscopy are typically older and symptomatic, whereas *CDH1* carriers are generally young and undergo screening or surveillance endoscopy.

Postoperative morbidity post gastrectomy was considerable, with 30.4% of patients requiring further intervention within 90 days—most often endoscopic dilatation for anastomotic stricture, a commonly demonstrated complication in contemporary HDGC series post total gastrectomy [29, 30].

Two patients (2.9%) developed an anastomotic leak and there were no procedure-related deaths, consistent with previous series reporting uncommon major surgical complications and procedure-related death [9, 31–34]. Patients should be counselled accordingly that although mortality is rare and invasive malignancy beyond pT1a is infrequent, up front gastrectomy remains a life-altering operation associated with meaningful functional consequences.

With respect to anatomical distribution, some studies have described a distal predominance of SRCC particularly within the antrum and transitional zone [14, 20, 23]. In contrast, our cohort showed a higher frequency of proximal involvement, with 39.4% of lesions in the fundus and 33% in the antrum, with some literature having a similar finding of fundus-predominant distribution in their cohorts [37], suggesting that proximal involvement may be more common than previously recognised. This variation may reflect true biological heterogeneity or differences in pathological sectioning and reporting. Our findings support the importance of systematic sampling across all gastric regions during endoscopic surveillance and in-depth examination of the entire surgical pathology specimen.

Gastric cancer risk assessment in *CDH1* carriers identified through lobular breast cancer remains an area of evolving clinical importance. Increasingly, *CDH1* carriers are identified through breast cancer–focused genetic testing [35], raising important questions about gastric cancer risk in such families [7, 36]. In our cohort, 39% of patients reported a family history of LBC and 10.7% had a personal history of LBC.

While SRCC was frequently identified in patients with a personal history of LBC or LCIS, no cases of invasive disease beyond pT1a were observed, suggesting that breast phenotype alone did not predict more advanced gastric pathology. These findings suggest that while gastric cancer risk remains clinically relevant in breast-presenting *CDH1* families [38, 39]. LBC phenotype may not correlate with an increased risk of invasive gastric cancer. Accordingly, LBC patients and families should undergo gastric risk assessment and structured surveillance counselling, with decisions regarding prophylactic gastrectomy individualised according to family history, age, and patient preference [2].

This study has several important strengths. With 82 *CDH1* carriers, 136 EGDs and 69 gastrectomies performed over 15 years, this represents a substantial single-center surveillance cohort providing sufficient data for meaningful analysis. The high proportion of patients proceeding to gastrectomy allows robust pathological correlation, and the 15-year span reflects evolving clinical practice from routine early risk reducing surgery toward more individualised surveillance strategies.

Several limitations should be acknowledged. As a retrospective single-centre study, our findings may not be generalisable. Many patients underwent only one EGD prior to up front risk reducing surgery, limiting comparability with contemporary structured surveillance cohorts. Use of surgical pathology as the reference standard may overestimate the clinical significance of microscopic SRCC, which may not inevitably progress to invasive cancer. Additionally, histopathological analysis of gastrectomy specimens may have been insufficient at times. Finally, relatively short surveillance follow-up (median 14.5 months) restricts assessment of long-term progression risk in patients managed non-operatively.

## Conclusions

In this large single-centre cohort, overall endoscopic SRCC detection sensitivity was 67.9%, with most lesions identified at the first examination and predominantly through systematic random biopsies. Multifocal SRCC was often detected on surgical pathology following gastrectomy, however invasive malignancy beyond pT1a was uncommon.

Our findings support growing recognition that many intramucosal, SRCC lesions may be more indolent than previously assumed, with an ongoing paradigm shift away from total gastrectomy [40]. In carefull4y counselled patients—particularly those without a strong family history of early diffuse gastric cancer—repeat endoscopy within a structured surveillance framework may be reasonable rather than reflex progression to immediate risk reducing gastrectomy. Surveillance represents a valid option for selected patients, provided its limitations are clearly communicated and structured follow-up is maintained. Continued prospective study with longer follow-up will be essential to refine progression risk estimates and information guidelines regarding surveillance and risk-reducing surgery in *CDH1* carriers.

## Data Availability

The datasets generated and/or analysed during the current study are not publicly available due to institutional and ethical restrictions but are available from the corresponding author on reasonable request.
